# Hepatitis C infection and complication rates after total shoulder arthroplasty in United States veterans

**DOI:** 10.1016/j.jseint.2021.02.009

**Published:** 2021-04-20

**Authors:** Favian Su, Charles J. Cogan, Ilya Bendich, Ning Zhang, Mary A. Whooley, Alfred C. Kuo

**Affiliations:** a Department of Orthopaedic Surgery, University of California, San Francisco, San Francisco, CA, USA; b Department of Medicine, University of California, San Francisco, San Francisco, CA, USA; c Veterans Affairs Quality Enhancement Research Initiative, San Francisco, CA, USA; d Department of Epidemiology and Biostatistics, University of California, San Francisco, San Francisco, CA, USA; e Orthopedic Surgery Section, San Francisco Veterans Affairs Health Care System, San Francisco, CA, USA

**Keywords:** Hepatitis C, Total shoulder arthroplasty, Veterans, Complications, Direct-acting antiviral, Surgical outcomes

## Abstract

**Background:**

Few studies have evaluated the effect of hepatitis C (HCV) on primary total shoulder arthroplasty (TSA). Our purpose was to determine if HCV infection is associated with increased complication rates after TSA in United States (US) veterans and, secondarily, to determine if preoperative HCV treatment with direct-acting antivirals (DAAs) affects postoperative complication rates.

**Methods:**

US Department of Veterans Affairs (VA) data sets were used to retrospectively identify patients without HCV, patients with untreated HCV, and patients with HCV treated with DAAs who underwent TSA from 2014 to 2019. Medical and surgical complications were assessed using International Classification of Diseases codes. Complication rates between patients with HCV (treated and untreated) and patients without HCV and between HCV-treated patients and HCV-untreated patients were compared at 90 days and 1 year after surgery.

**Results:**

We identified 5774 primary TSAs that were performed at VA hospitals between 2014 and 2019. A minority (9.5%) of TSA patients had HCV, 23.4% of whom were treated preoperatively with DAAs. On multivariate analysis, HCV patients had increased odds of 1-year medical complications (odds ratio, 1.39; 95% confidence interval, 1.06-1.81, *P* = .016), when compared with patients without HCV. No statistically significant difference in complication rates was observed between HCV-treated and HCV-untreated patients.

**Discussion:**

US veterans with a history of HCV are at an increased risk of developing medical but not surgical complications within the first year after TSA. Larger studies are necessary to evaluate the effects of DAA treatment on complication rates.

Anatomic and reverse total shoulder arthroplasty (TSA) are safe and effective procedures for treating various shoulder pathologies, including osteoarthritis, rotator cuff arthropathy, and fracture.^25^ A number of patient factors are associated with increased risks of medical and surgical complications following TSA, including diabetes, renal failure, depression, smoking, human immunodeficiency virus (HIV) infection, and hepatitis C virus (HCV) infection.^3^^,^^9^^,^^15^ Studies of hip and knee arthroplasties suggest that preoperative modification of some of these risk factors may lead to decreased complication rates after surgery.^5^^,^^18^ Treatment of HCV with direct-acting antivirals (DAA) before hip and knee arthroplasty is associated with lower postoperative complication rates.^5^

The prevalence of HCV infection among patients undergoing orthopedic surgery has been estimated to range from 3% to 8%.^8^^,^^11^ The United States veteran population, however, has been reported to have greater than 16 times the rate of HCV infection compared to the general population. In 2014, the Department of Veterans Affairs (VA) launched a campaign to identify and treat veterans with HCV, and recent data from one center suggests that the use of DAA therapy has decreased the prevalence of HCV in patients undergoing arthroplasty to as low as 0.4%.^20^^,^^23^ Both medical and surgical complications are well documented for patients with HCV undergoing hip and knee arthroplasty, including acute postoperative infection, mechanical complications, and general medical complications.^5^^,^^6^^,^^11^^,^^22^ Only one study to date has assessed HCV as a risk factor for postoperative complications in shoulder arthroplasty. Medicare patients with HCV undergoing TSA were found to be at significantly higher risk for both medical and surgical complications.^9^ The purpose of our study was to determine if HCV infection is also associated with increased medical and surgical complication rates after TSA in US veterans. Secondarily, we sought to determine if preoperative HCV treatment with DAAs affected postoperative complication rates. We hypothesized that HCV patients would have higher rates of medical and surgical complications after TSA compared with patients without HCV. We further hypothesized that HCV patients who received preoperative DAA treatment would experience fewer postoperative medical and surgical complications after TSA compared with untreated HCV patients.

## Methods

### TSA data set

**Figure 1:**
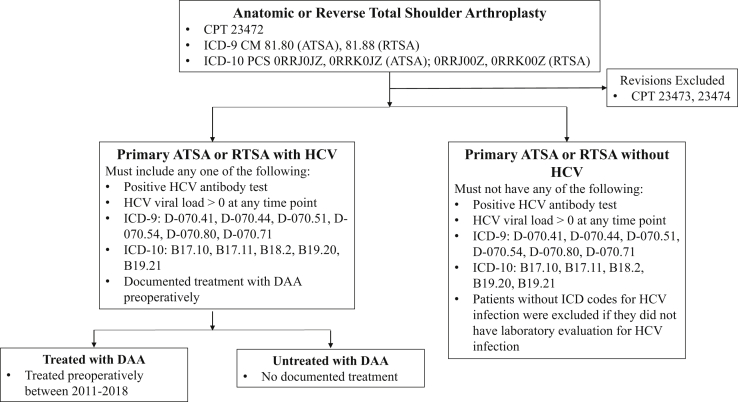
Cohort selection. *ICD*, International Classification of Diseases; *CPT*, Common Procedural Terminology; *ATSA*, anatomic total shoulder arthroplasty; *RTSA*, reverse total shoulder arthroplasty; *HCV*, hepatitis C virus; *DAA*, direct-acting antiviral.

This study was performed after obtaining institutional review board approval. The VA Corporate Data Warehouse (CDW) was used to identify a cohort of patients who underwent TSA (both anatomic and reverse) at US Veterans Health Administration (VHA) hospitals between January 1, 2014 and September 30, 2019. This study interval was selected because the VHA embarked on a system-wide campaign to curatively treat all US veterans with HCV infection in 2014. Common Procedural Terminology (CPT) codes were used to identify patients who underwent primary total shoulder arthroplasty (23472). Since CPT codes do not differentiate between anatomic (ATSA) and reverse TSA (RTSA), International Classification of Diseases, Ninth and Tenth Revision (ICD-9 and ICD-10) codes were used to further subclassify patients into ATSA and RTSA (Fig. 1).

### Identifying TSA patients with HCV

Patients with laboratory evidence of HCV infection (positive HCV antibody, RNA, or viral load) were considered to have HCV. Additionally, ICD-9 (070.41, 070.44, 070.51, 070.54, 070.70, 070.71) and ICD-10 (B17.10, B17.11, B18.2, B19.20, B19.21) codes for HCV were used to identify patients with HCV. To account for patients who may have been diagnosed with HCV outside of the VHA system but received HCV treatment at the VHA, patients were also considered to be HCV positive if they had documented DAA treatment (daclatasvir, dasabuvir/ombitasvir/paritaprevir/ritonavir, elbasvir/grazoprevir, ledipasvir/sofosbuvir, ombitasvir/paritaprevir/ritonavir, simeprevir, sofosbuvir, sofosbuvir/velpatasvir, sofosbuvir/velpatasvir/voxilaprevir). These treatments are not used for alternative diagnoses. Patients with negative laboratory tests for HCV who had no ICD codes for HCV and no HCV-specific treatment were classified as HCV-free. Patients without ICD codes for HCV and without HCV-specific treatment were excluded if they had no laboratory testing for HCV.

### Identifying HCV-treated and HCV-untreated patients

Patients treated with DAA-based therapies before TSA were classified as “treated.” Patients treated with older therapies, including ribavirin and interferon, were excluded from the analysis due to lower overall cure rates (around 50%) and higher complication rates relative to DAA-based therapies, which have cure rates over 95%.^4^^,^^17^ An undetectable HCV viral load was not used to confirm successful treatment.

ICD-9 and ICD-10 codes were used to identify preoperative comorbidities and to calculate the Charlson Comorbidity Index (CCI) for each patient.^24^ A comorbid condition was considered present if it was recorded in the patient’s record during one inpatient or 2 outpatient encounters between January 1, 2014 and the date of surgery.

### Outcome ascertainment

ICD codes were also used to identify medical and surgical complications within 90 days and 1 year after surgery (Table I) from inpatient and outpatient files in the VA CDW and from purchased care files (for care provided at a non-VA facility).

**Table I:** ICD-9 and ICD-10 codes for medical and surgical complications.

Postoperative medical complications	ICD-9 codes	ICD-10 codes
Acute myocardial infarction	410.00, 410.01, 410.10, 410.11, 410.20, 410.21, 410.30, 410.31, 410.40, 410.41, 410.50, 410.51, 410.60, 410.61, 410.70, 410.71, 410.80, 410.81, 410.90, 410.91, 997.1	I21.09, I21.11, I21.19, I21.29, I21.4, I21.3, I21.9, I21.A1, I21.A9, I97.710, I97.790
Pulmonary embolism	415.11, 415.12, 415.13, 415.19, 416.2	I26.90, I26.99, I26.92, I26.99, I27.82
Pneumonia	480-480.9, 481, 482-482.9, 483, 483.1, 483.8, 484, 484.1, 484.3, 484.5-484.8, 485, 486, 487, 507	J12.0, J12.1, J12.9, J13.0, J15.0, J15.4, J15.6, J15.9, J16.0, J16.8, J18.0, J18.1, J18.9, J25.0, A37.91
Deep vein thrombosis	453.4, 453.41, 453.42, 453.9	I82.419, I82.429, I82.449, I82.499, I82.4z9, I82.91, I82.439, I82.4Y9
Sepsis	995.91, 995.92	A40.9, A40.89, A41.9, R65.20
Urinary tract infection	599, 997.5	N99.89
Cerebrovascular accident	433.01, 433.11, 433.21, 433.31, 433.81, 433.91, 434.01, 434.11, 434.91, 437.1, 997.01, 997.02	I63.22, I63.139, I63.239, I63.019, I63.119, I63.219, I63.59, I63.30, I63.40, I63.50, I63.59, I67.81, I67.82, I67.89, G97.81, G97.82, I97.811, I97.821
Acute kidney injury	584, 584.5, 584.6, 584.7, 584.8, 584.9, 586	N17.0, N17.1, N17.2, N17.8, N17.9, N19
Postoperative surgical complications	ICD-9 codes	ICD-10 codes
Wound disruption/dehiscence	998.30, 998.32	T81.30XA, T81.31XA, T81.32XA
Implant infection	996.66, 996.67, 996.69	T84.50XA, T84.59XA
Mechanical complication (dislocation, loosening)	996.42, 831.00-831.03, 996.41	T84.028A, T84.029A, S43.00, S43.01, S43.02, S43.03, S43.08, T84.038A, T84.039A

*ICD*, International Classification of Diseases.

### Statistical analysis

Analysis of variance, Fisher’s exact test, and chi-squared analyses were utilized to compare differences in demographics and complication rates at 90 days and at 1 year after surgery among patients without HCV, untreated HCV patients, and treated HCV patients. Multivariate logistic regression was performed to identify odds ratios (ORs) of implant infection, any medical complication, and any surgical complication at 90 days and at 1 year. Comparisons were made between patients with (treated or untreated) HCV and patients without HCV, as well as between HCV treated and HCV untreated patients. In addition to HCV status, the regression analysis adjusted for all variables that were significantly different between groups, including age, gender, HIV status, hepatitis B virus infection, history of smoking, and CCI.

## Results

We identified 5774 primary TSAs (1680 ATSA, 1367 RTSA, 2727 unspecified) that were performed at US VHA hospitals between 2014 and 2019 that met our inclusion and exclusion criteria. Of these, 548 patients (9.5% of all TSA) were identified to have been infected with HCV. One hundred twenty-eight patients, or 23.4% of TSA patients with HCV, had their HCV treated preoperatively with DAA-based therapies (Table II). Patients with HCV were significantly younger (*P* < .001). The average age of patients without HCV was 67.3 ± 7.8 years, the average age of untreated HCV patients was 64.1 ± 5.8 years, and the average age of treated HCV patients was 64.0 ± 5.2 years. Patients with HCV had an increased rate of HIV infection (1.9% vs. 0.2%, *P* < .001), hepatitis B infection (1.1% vs. 0.2%, *P* < .001), and history of smoking (75.7% vs. 64.5%, *P* < .001) compared to patients without HCV. Additionally, patients with HCV had a significantly higher CCI than patients without HCV (*P* < .001). Patients with treated HCV had an increased rate of concomitant HIV infection (4.7% vs. 1.0%, *P* = .006), history of smoking (84.4% vs. 73.1%, *P* = .009), and significantly higher CCI (*P* < .001) than patients with untreated HCV. Interestingly, diabetes mellitus was significantly more common in patients without HCV compared to patients with treated and untreated HCV (27.9% vs. 19.8% and 20.3%, respectively, *P* < .001).

**Table II:** Patient demographics.

	Total shoulder arthroplasty						Anatomic total shoulder arthroplasty						Reverse total shoulder arthroplasty					
	Total no. of surgeries (n = 5774)	TSA without HCV (n = 5226)	TSA with untreated HCV (n = 420)	TSA with treated HCV (n = 128)	*P* value Patients with HCV vs. without HCV	*P* value Patients with untreated HCV vs. treated HCV	Total no. of surgeries (n = 1680)	ATSA without HCV (n = 1513)	ATSA with untreated HCV (n = 143)	ATSA with treated HCV (n = 24)	*P* value Patients with HCV vs. without HCV	*P* value Patients with untreated HCV vs. treated HCV	Total no. of surgeries (n = 1367)	RTSA without HCV (n = 1251)	RTSA with untreated HCV (n = 87)	RTSA with treated HCV (n = 29)	*P* value Patients with HCV vs. without HCV	*P* value Patients with untreated HCV vs. treated HCV
Age (yr)	67.0 ± 7.7	67.3 ± 7.8	64.1 ± 5.8	64.0 ± 5.2	**<.001**	.91	65.3 ± 7.8	65.5 ± 8.0	63.6 ± 5.6	62.6 ± 5.7	**<.001**	.62	68.6 ± 7.0	69.0 ± 7.1	64.1 ± 4.6	64.3 ± 5.0	**<.001**	.84
Gender																		
Female	322	5.7%	4.0%	3.9%	.09	.94	98	6.2%	2.8%	0.0%	**.05**	.41	69	5.2%	4.6%	.0%	.41	.24
Male	5452	94.3%	96.0%	96.1%			1582	93.8%	97.2%	100.0%			1298	94.8%	95.4%	100.0%		
Year of surgery																		
2014	898	15.4%	21.9%	0.0%	.84	**<.001**	445	26.0%	36.4%	0.0%	.26	**<.001**	292	21.7%	23.0%	.0%	.34	.06
2015	1109	19.1%	23.1%	10.2%			437	25.9%	28.7%	16.7%			373	27.3%	27.6%	27.6%		
2016	1153	20.0%	19.5%	21.1%			256	15.6%	11.9%	12.5%			188	13.4%	17.2%	17.2%		
2017	1109	19.2%	14.5%	32.8%			207	12.8%	6.3%	16.7%			203	14.5%	14.9%	27.6%		
2018	1303	22.7%	18.3%	32.8%			295	17.4%	14.0%	50.0%			262	19.2%	16.1%	27.6%		
2019	202	3.6%	2.6%	3.1%			40	2.3%	2.8%	4.2%			49	3.8%	1.1%	.0%		
HIV	22	.2%	1.0%	4.7%	**<.001**	**.006**	8	0.3%	0.7%	12.5%	**<.001**	**<.001**	5	.3%	1.1%	.0%	.35	.56
Hepatitis B	14	.2%	1.4%	.0%	**<.001**	.17	6	0.2%	2.1%	0.0%	**<.001**	.47	2	.1%	1.1%	.0%	**.04**	.56
Smoking	3785	64.5%	73.1%	84.4%	**<.001**	**.009**	1055	61.5%	74.8%	75.0%	**<.001**	.99	942	67.9%	77.0%	86.2%	**.01**	.29
Diabetes	1567	27.9%	19.8%	20.3%	**<.001**	.89	436	26.6%	20.3%	16.7%	**.05**	.68	410	30.9%	19.5%	20.7%	**.01**	.89
BMI ≥ 40	314	5.5%	5.2%	1.6%	.25	.08	110	6.7%	4.9%	4.2%	.33	.88	77	5.9%	3.4%	.0%	.14	.31
Charlson score																		
0	2431	43.6%	33.3%	7.8%	**<.001**	**<.001**	744	45.8%	34.3%	8.3%	**<.001**	**<.001**	513	39.0%	26.4%	6.9%	**<.001**	**.05**
1-3	2786	47.0%	56.9%	71.1%			811	47.1%	58.7%	62.5%			688	48.9%	63.2%	72.4%		
≥ 4	557	9.4%	9.8%	21.1%			125	7.1%	7.0%	29.2%			166	12.1%	10.3%	20.7%		

*TSA*, total shoulder arthroplasty; *HCV*, hepatitis C virus; *ATSA*, anatomic total shoulder arthroplasty; *RTSA*, reverse total shoulder arthroplasty; *HIV*, human immunodeficiency virus; *BMI*, body mass index.

Bold denotes significance.

**Figure 2:**
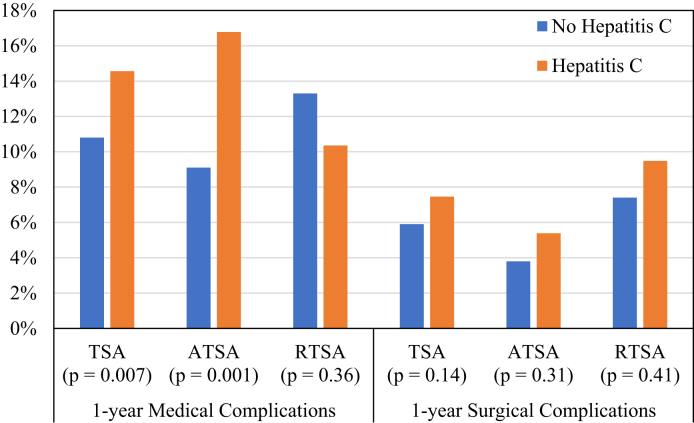
Medical and surgical complication rates in patients with and without HCV 1 year after total shoulder arthroplasty (TSA), anatomic total shoulder arthroplasty (ATSA), or reverse total shoulder arthroplasty (RTSA).

We next calculated TSA 90-day and 1-year medical and surgical complication rates (Table III and Fig. 2). The most frequently coded medical complication was acute kidney injury (AKI), accounting for 44.1% and 45.0% of all medical complications at 90 days and 1 year, respectively. At 90 days after surgery, urinary tract infections were significantly more common in patients without HCV compared to patients with HCV (0.7% vs. 0.0%, *P* = .05). At 1 year, patients with HCV had a higher rate of AKI (6.8% vs. 4.8%, *P* = .05), pneumonia (5.0% vs. 2.9%, *P* = .009), acute myocardial infarction (2.6% vs. 1.5%, *P* = .05), and sepsis (2.7% vs. 1.4%, *P* = .01) compared to patients without HCV. The rate of medical complications was not significantly different between treated and untreated HCV groups (14.0% vs. 16.4%, *P* = .51).

**Table III:** TSA complication rates at 90 d and 1 yr.

	No. of patients with complications	TSA without HCV (n = 5226)	TSA with untreated HCV (n = 420)	TSA with treated HCV (n = 128)	*P* value Patients with HCV vs. without HCV	*P* value Patients with untreated HCV vs. treated HCV
90-d medical complications	324	5.6%	4.5%	8.6%	.88	.08
Acute kidney injury	143	2.5%	1.9%	4.7%	.90	.08
Acute myocardial infarction	39	.6%	1.2%	1.6%	.07	.74
Cerebrovascular accident	15	.3%	.2%	.0%	.71	.58
Deep vein thrombosis	27	.5%	.2%	.0%	.30	.58
Pneumonia	65	1.1%	1.4%	.8%	.72	.57
Pulmonary embolism	40	.7%	.2%	.8%	.33	.37
Sepsis	32	.6%	.2%	1.6%	.98	.08
Urinary tract infection	37	.7%	.0%	.0%	**.05**	N/A
90-d surgical complications	211	3.6%	4.3%	3.9%	.48	.85
Wound disruption	25	.4%	.2%	1.6%	.67	.08
Implant infection	77	1.3%	1.9%	1.6%	.29	.80
Mechanical complication	131	2.2%	3.3%	.8%	.44	.12
1-yr medical complications	644	10.8%	14.0%	16.4%	**.007**	.51
Acute kidney injury	290	4.8%	6.2%	8.6%	**.05**	.34
Acute myocardial infarction	90	1.5%	2.9%	1.6%	**.05**	.42
Cerebrovascular accident	46	.7%	1.2%	1.6%	.18	.74
Deep vein thrombosis	41	.7%	.7%	.8%	.95	.94
Pneumonia	178	2.9%	4.8%	5.5%	**.009**	.75
Pulmonary embolism	66	1.2%	.5%	.8%	.17	.68
Sepsis	88	1.4%	3.1%	1.6%	**.01**	.35
Urinary tract infection	44	.8%	.5%	.0%	.26	.43
1-yr surgical complications	350	5.9%	7.6%	7.0%	.14	.82
Wound disruption	41	.7%	.2%	1.6%	.63	.08
Implant infection	128	2.2%	3.1%	1.6%	.38	.35
Mechanical complication	231	3.9%	5.7%	3.9%	.10	.42

*TSA*, total shoulder arthroplasty; *HCV*, hepatitis C virus.

Bold denotes significance.

The complication rates for ATSA and RTSA are displayed in Tables IV and V, respectively. Among patients who underwent ATSA, those with HCV had a significantly higher rate of overall medical complication (16.8% vs. 9.1%, *P* = .001), AKI (8.4% vs. 3.8%, *P* = .006), pneumonia (5.4% vs. 1.7%, *P* = .001), and sepsis (3.0% vs. 0.7%, *P* = .002) compared to those without HCV. Only significantly higher rates of sepsis were observed for patients with HCV undergoing RTSA compared to those without HCV (4.3% vs. 1.7% *P* = .05).

**Table IV:** ATSA complication rates at 90 d and 1 yr.

	No. of patients with complications (n = 1680)	ATSA without HCV (n = 1513)	ATSA with untreated HCV (n = 143)	ATSA with treated HCV (n = 24)	*P* value Patients with HCV vs. without HCV	*P* value Patients with untreated HCV vs. treated HCV
90-d medical complications	76	4.6%	3.5%	8.3%	.83	.27
Acute kidney injury	26	1.6%	1.4%	.0%	.70	.56
Acute myocardial infarction	13	.7%	1.4%	.0%	.51	.56
Cerebrovascular accident	3	.2%	.0%	.0%	.56	N/A
Deep vein thrombosis	7	.5%	.0%	.0%	.38	N/A
Pneumonia	14	.9%	.0%	4.2%	.73	**.01**
Pulmonary embolism	10	.6%	.7%	.0%	.99	.68
Sepsis	3	.1%	.0%	4.2%	.18	**.01**
Urinary tract infection	15	1.0%	.0%	.0%	.20	N/A
90-d surgical complications	36	2.2%	1.4%	.0%	.37	.56
Wound disruption	4	.3%	.0%	.0%	.51	N/A
Implant infection	20	1.3%	.7%	.05%	.46	.68
Mechanical complication	15	.9%	.7%	.0%	.67	.68
1-yr medical complications	165	9.1%	17.5%	12.5%	**.001**	.55
Acute kidney injury	72	3.8%	9.1%	4.2%	**.006**	.42
Acute myocardial infarction	31	1.7%	3.5%	.0%	.25	.35
Cerebrovascular accident	15	.8%	1.4%	4.2%	.19	.34
Deep vein thrombosis	12	.7%	.7%	.0%	.85	.68
Pneumonia	34	1.7%	4.9%	8.3%	**.001**	.49
Pulmonary embolism	19	1.2%	.7%	.0%	.49	.68
Sepsis	15	.7%	2.8%	4.2%	**.002**	.72
Urinary tract infection	19	1.2%	.7%	.0%	.49	.68
1-yr surgical complications	66	3.8%	6.3%	.0%	.31	.21
Wound disruption	7	.5%	.0%	.0%	.38	N/A
Implant infection	31	1.9%	2.1%	.0%	.96	.47
Mechanical complication	35	1.9%	4.9%	.0%	**.04**	.27

*ATSA*, anatomic total shoulder arthroplasty; *HCV*, hepatitis C virus.

Bold denotes significance.

**Table V:** RTSA complication rates at 90 d and 1 yr.

	No. of patients with complications (n = 1367)	RTSA without HCV (n = 1251)	RTSA with untreated HCV (n = 87)	RTSA with treated HCV (n = 29)	*P* value Patients with HCV vs without HCV	*P* value Patients with untreated HCV vs. treated HCV
90-d medical complications	91	7.1%	2.3%	.0%	**.03**	.41
Acute kidney injury	43	3.4%	1.1%	.0%	.14	.56
Acute myocardial infarction	8	.6%	.0%	.0%	.39	N/A
Cerebrovascular accident	6	.4%	1.1%	.0%	.47	.56
Deep vein thrombosis	12	1.0%	.0%	.0%	.29	N/A
Pneumonia	15	1.2%	.0%	.0%	.24	N/A
Pulmonary embolism	12	1.0%	.0%	.0%	.29	N/A
Sepsis	10	.8%	.0%	.0%	.33	N/A
Urinary tract infection	11	.9%	.0%	.0%	.31	N/A
90-d surgical complications	64	4.6%	8.0%	.0%	.47	.12
Wound disruption	4	.3%	.0%	.0%	.54	N/A
Implant infection	17	1.2%	2.3%	.0%	.63	.41
Mechanical complication	49	3.4%	8.0%	.0%	.14	.12
1-yr medical complications	179	13.3%	11.5%	6.9%	.36	.48
Acute kidney injury	83	6.4%	3.4%	.0%	.10	.31
Acute myocardial infarction	22	1.7%	1.1%	.0%	.50	.56
Cerebrovascular accident	15	1.0%	1.1%	3.4%	.50	.41
Deep vein thrombosis	14	1.1%	.0%	.0%	.25	N/A
Pneumonia	50	3.6%	4.6%	3.4%	.70	.79
Pulmonary embolism	16	1.2%	1.1%	.0%	.75	.56
Sepsis	26	1.7%	5.7%	.0%	**.05**	.19
Urinary tract infection	12	1.0%	.0%	.0%	.29	N/A
1-yr surgical complications	103	7.4%	11.5%	3.4%	.41	.20
Wound disruption	8	.6%	.0%	.0%	.39	N/A
Implant infection	36	2.5%	5.7%	.0%	.24	.19
Mechanical complication	73	5.2%	8.0%	3.4%	.44	.40

*RTSA*, reverse total shoulder arthroplasty; *HCV*, hepatitis C virus.

Bold denotes significance.

The most frequently coded surgical complications were mechanical in nature, accounting for 62.1% and 66.0% of the surgical complications at 90 days and 1 year. Among all TSAs, there were no significant differences in wound disruption, implant infection, or mechanical complications among the groups at 90 days and at 1 year. However, for the ATSA subgroup, patients with HCV had a significantly higher rate of mechanical complications than patients without HCV at 1 year (4.9% vs. 1.9%, *P* = .04). Although the ATSA HCV-treated cohort had no mechanical complications at 1 year, this was not significant compared to the untreated cohort (*P* = .27).

Multivariate logistic analysis was performed to evaluate the associations between HCV status and complication rates (Table VI). At 90 days, there was no difference in implant infection rates, any medical complication rate, or any surgical complication rate between patients with and without HCV. However, at 1 year, patients with HCV undergoing TSA or ATSA had a significantly increased odds of developing a medical complication compared to patients without HCV (TSA: OR = 1.39 [95% confidence interval: 1.06-1.81], *P* = .016; ATSA: OR = 2.02 [95% confidence interval: 1.27-3.21], *P* = .030). There was no difference in implant infection rates, medical complication rates, or surgical complication rates between patients in the HCV-treated and HCV-untreated cohorts.

**Table VI:** Multivariate analysis of TSA, ATSA, and RTSA complications at 90 d and 1 yr.

	HCV infection vs. no HCV infection∗		HCV untreated vs. HCV treated†	
	OR (95% CI)	*P* value	OR (95% CI)	*P* value
TSA 90 d				
Implant infection	1.25 (0.63-2.48)	.533	1.11 (0.23-5.31)	.895
Any medical complication	0.96 (0.64-1.43)	.825	.74 (0.38-1.44)	.376
Any surgical complication	1.09 (0.69, 1.71)	.707	.98 (0.38-2.52)	.960
TSA 1 yr				
Implant infection	1.18 (0.68-2.06)	.559	1.72 (0.38-7.80)	.481
Any medical complication	1.39 (1.06-1.81)	**.016**	.81 (0.49-1.33)	.397
Any surgical complication	1.16 (0.82-1.64)	.399	.92 (0.45-1.88)	.820
ATSA 90 d				
Implant infection	.36 (0.05-2.74)	.322	N/A	N/A
Any medical complication	.88 (0.39-2.01)	.768	.80 (0.12-5.24)	.812
Any surgical complication	.45 (0.11-1.91)	.277	N/A	N/A
ATSA 1 yr				
Implant infection	.84 (0.25-2.84)	.778	N/A	
Any medical complication	2.02 (1.27-3.21)	**.003**	1.83 (0.35-9.57)	.472
Any surgical complication	1.26 (0.60-2.64)	.545	N/A	
RTSA 90 d				
Implant infection	1.19 (0.26-5.5)	.827	N/A	N/A
Any medical complication	.25 (0.06-1.05)	.059	N/A	N/A
Any surgical complication	1.27 (0.54-2.95)	.584	N/A	N/A
RTSA 1 yr				
Implant infection	1.48 (0.54-4.06)	.444	2.45 (0.56-10.70)	.234
Any medical complication	.76 (0.40-1.46)	.416	2.36 (0.31-17.93)	.407
Any surgical complication	1.20 (0.60-2.37)	.610	2.37 (0.70-8.09)	.168

*TSA*, total shoulder arthroplasty; *ATSA*, anatomic total shoulder arthroplasty; *RTSA*, reverse total shoulder arthroplasty; *HCV*, hepatitis C virus; *OR*, odds ratio; *CI*, confidence interval.

Bold denotes significance.

∗ Reference group is patients without HCV.

† Reference group is patients with treated HCV.

## Discussion

Despite the high prevalence of HCV among US veterans, few studies have evaluated the effect of HCV on primary TSA.^2^ In this study, patients with HCV were found to have an increased risk of medical complications 1 year after TSA compared to those without. Although a subgroup analysis of patients undergoing ATSA showed a significantly higher 1-year rate of mechanical complications with HCV on univariate analysis, there was no other difference in surgical complications between patients with and without HCV. Moreover, preoperative HCV treatment with a DAA was not associated with lower complication rates.

This study demonstrated that patients with HCV were significantly more likely to develop AKI, pneumonia, acute myocardial infarction, and sepsis compared to controls. Furthermore, the incidence of major complications, such as myocardial infarction and sepsis, was not inconsequential as the overall risk was greater than 2.6% at 1 year. These findings extend those of a Medicare database study involving 22,968 TSA patients, which found a significantly increased systemic medical complication rate of 5.9% in patients with HCV compared to 4.6% in patients without HCV at 90-days.^9^

The association between HCV infection status and the increased risk of medical complications has also been well documented in the hip and knee arthroplasty literature.^5^^,^^6^^,^^11^ Bendich et al reported that US veterans with HCV had higher rates of septic complications at 90 days and 1 year after hip and knee total joint arthroplasty (TJA) compared to those without HCV.^5^ Similarly using the National Inpatient Sample database, Issa et al found that when compared to matched controls, TJA patients with HCV had a 15% increased risk of a medical complication.^11^ Best et al used the National Hospital Discharge Survey to demonstrate that HCV patients had twice the odds of suffering a general medical complication compared to uninfected patients.^6^ The underlying reasons for increased medical complications among HCV patients may be related to extrahepatic manifestations of the disease. Prior studies have hypothesized that a combination of cryoglobulin small vessel vasculitis, impaired lymphoproliferation, and disrupted kidney and hematologic function may predispose HCV patients to the increased risk for several of the medical complications demonstrated in this study.^11^^,^^22^

Although HCV infection was associated with an increased rate of medical complications, it was not associated with an increased rate of implant infection or surgical complications after TSA. On subgroup analysis, HCV patients undergoing ATSA had a significantly higher rate of mechanical complications compared to uninfected patients at 1 year. These findings differ from those of a prior national database study, which showed not only increased of mechanical complications, such as dislocation, but also increased rates of infection, revision surgery, stiffness, and fracture among HCV patients undergoing TSA.^9^ These discrepancies may be due to significant differences between our cohorts, such as increased percentage of female patients, prevalence of diabetes, and a larger sample size in the previous study.^10^^,^^15^^,^^16^ Our results are also in contrast to the overall trends in the hip and knee arthroplasty literature, in which HCV patients had increased surgical complications, including prosthetic joint infection, mechanical complications, and postoperative bleeding, compared with matched controls.^5^^,^^6^^,^^11^^,^^12^^,^^21^^,^^22^ Many of these studies utilized national databases with much larger sample sizes, which may suggest that our study may have been underpowered to detect a difference in surgical complications.

To the best of our knowledge, this is the first study to examine the effects of preoperative HCV treatment with DAA on rates of common medical and surgical complications after TSA. Patients who had undergone HCV treatment preoperatively had a 1.5% and 1.8% lower rate of implant infection and mechanical complication, respectively, compared to untreated patients 1 year after TSA. The rates of implant infection and mechanical complication were also similar in TSA patients without HCV and those with treated HCV. Although these differences did not reach statistical significance, the differences were in the expected direction, and the magnitude of these differences was similar to the results of prior studies evaluating the effect of HCV treatment in hip and knee arthroplasty. Bendich et al showed that preoperative DAA treatment was associated with significantly reduced rates of 1-year implant infectious and mechanical complications (1.8% and 0.8% reductions, respectively), compared to untreated patients.^5^ The trend toward decreased infection and mechanical complication rate after DAA treatment may be related to the improvement in multisystem function associated with sustained virologic response. Prior studies evaluating the effects of DAA therapy have demonstrated a decreased rate incidence of mixed cryoglobulinemia, lymphoproliferative disorders, diabetes, and cardiovascular disease after treatment.^19^ Despite the benefits in treating hepatic and extrahepatic manifestations, there is currently insufficient evidence to recommend preoperative DAA treatment in HCV patients undergoing TSA. However, future studies are warranted on this topic.

Interestingly, our results also demonstrated higher rates of AKI in HCV patients who received treatment preoperatively compared to untreated patients. Although DAAs have a relatively safe adverse effect profile, Brown et al reported that nearly 20% of patients treated with DAA had AKI during DAA therapy.^7^ Furthermore, prior studies have found no significant difference in renal function between untreated and treated HCV patients, suggesting that viral eradication may not be associated with improvement in renal disease progression.^1^ This stresses the importance of monitoring renal function in TSA patients in patients with HCV who recently received DAA therapy.

Another important finding in this study is the 9.5% prevalence of HCV in US veterans undergoing TSA in the past 5 years. This rate is much higher than the national prevalence of 1.3% and the 2014 US veteran prevalence of 6.1%.^2^^,^^8^ Furthermore, there was a decrease in the prevalence of HCV infected veterans undergoing TSA from 11.4% in 2014 to 6.5% in 2018. Recently, Shapiro et al reported a decrease in overall viremic prevalence from 53.1% in 2012 to 3.0% in 2019 among HCV patients undergoing total hip and knee arthroplasty at a single California VA medical center.^8^^,^^23^ The current study utilized a nationwide database, which may account for the difference in the rate of treated HCV patients. Nonetheless, their study underscores that the rates of HCV treatment among US veterans is lagging nationally.

Although this study utilized a large national database of a single population of patients with high HCV prevalence, it is not without limitations, including its retrospective nature. In addition, like other studies that analyze a VA patient cohort, there is a patient demographic bias that underrepresents female patients and young patients, limiting the generalizability of the findings. Second, HCV status and complications were identified using ICD-9 and ICD-10 codes, which may be inaccurate. However, prior studies evaluating the coding accuracy of ICD-9 codes in the VHA system showed a positive predictive value of 93% and negative predictive value of 92% in correctly identifying HCV status.^13^^,^^14^ We also cannot rule out potential cases of misclassification in which HCV patients were treated with DAA therapy that was not documented in the medical record. Another limitation is the relatively small sample size of HCV-treated and HCV-untreated patients. This study was likely underpowered to detect a difference in surgical complications as previously reported by larger database studies using the National Inpatient Sample.^9^ A post hoc power analysis showed that 19,623 patients would be needed to demonstrate a significant difference in implant infection rates between patients with and without HCV. Next, this study did not divide HCV patients into those with and without cirrhosis. It has been previously shown that cirrhosis is an independent risk factor for increased postoperative complications after TJA.^21^ However, this study investigated only patients who received arthroplasty, and patients who were ill due to cirrhosis may not have been TSA candidates. Furthermore, we did not include transfusion rates or revision rates, which are all factors that have been reported to be elevated in HCV TSA cohorts.^9^ Finally, we did not prove cure with DAA-based treatments of HCV. We did not view this as necessary as recent therapies have generated cure rates over 95%.^4^

## Conclusion

In summary, 9.5% of patients undergoing TSA in the US VHA system from 2014 to 2019 had HCV infection. Patients with HCV are at an increased risk of medical complications, including AKI, acute myocardial infarction, pneumonia, and sepsis, 1 year after TSA compared to uninfected patients. The overall risk for these complications is not inconsequential and surgeons should discuss this risk with patients and maintain vigilance for cardiovascular and infectious signs postoperatively. There was no significant difference in surgical complication rates between patients with and without HCV at 90 days and 1 year. At this time, there is insufficient evidence to recommend preoperative DAA treatment in HCV patients undergoing TSA to decrease medical or surgical complications, although there are other benefits of HCV treatment. Larger studies are needed in the future to evaluate the effect of HCV treatment on outcomes in TSA.

## Disclaimers:

*Funding:* This study was funded by the VA Quality Enhancement Research Initiative (VA 150HX003266) and a James O. Johnston Resident Research Grant.

*Conflicts of interest:* The authors, their immediate family, and any research foundation with which they are affiliated have not received any financial payments or other benefits from any commercial entity related to the subject of this article. This study was funded by the VA Quality Enhancement Research Initiative (VA 150HX003266) and a James O. Johnston Resident Research Grant.
